# Frequency-tunable acoustic absorption in anisotropic graphene aerogels *via* morphological engineering of internal barriers

**DOI:** 10.1039/d6ra01470d

**Published:** 2026-05-12

**Authors:** Jaeeun Jin, Jae Gyu Ahn, An So Yeon, Ryunkyong Lee, Taeyoung Park, Dong Ju Lee, Sung Ho Song

**Affiliations:** a Division of Advanced Materials Engineering, Center for Advanced Materials and Parts of Powders, Kongju National University Cheonan-si 31080 Republic of Korea shsong805@kongju.ac.kr; b Department of Urban, Energy, and Environmental Engineering, Chungbuk National University Cheongju 28644 Republic of Korea dongjulee@chungbuk.ac.kr

## Abstract

The development of high-efficiency acoustic attenuation media has been significantly advanced by the integration of graphene derivatives and carbon nanotubes, taking advantage of their superior chemical and mechanical attributes. Nevertheless, a primary obstacle in engineering these materials involves the precise morphological control of low-dimensional nanostructures—specifically regarding the prevention of random restacking and the achievement of uniform directional alignment within the porous framework. Furthermore, systematic investigations into the acoustic properties of pure graphene aerogels—specifically regarding how flake dimensions and loading influence the barrier effect and structural anisotropy—remain largely unexplored. To address these challenges, this research demonstrates a systematic approach to engineering anisotropic graphene-based aerogels featuring frequency-tunable acoustic absorption through synergistic modulation *via* bidirectional freeze-casting. By employing the Ice-Segregation-Induced Self-Assembly (ISISA) process, the study successfully fabricated highly ordered, vertically and horizontally aligned lamellar networks. Morphological investigations revealed a distinct structural divergence: aerogels based on large graphene flakes formed continuous, streamlined micro-channels with minimal structural resistance, whereas variants utilizing smaller flakes developed a high density of internal structural barriers, or septa. The superior performance of these structures is driven by intensified visco-thermal energy dissipation and multiple scattering effects, both of which are facilitated by increased tortuosity and internal air-flow resistance. Furthermore, by manipulating the spatial orientation of the framework relative to the incident sound vector, we proposed a mechanism to explain how the barrier effect sustains enhanced absorption in transverse modes compared to longitudinal ones. These findings establish a robust structure–property relationship between microscopic precursor dimensions and macroscopic hierarchical architecture, offering a new paradigm for high-performance, multifunctional carbon-based noise insulators.

## Abstract 2

The development of high-efficiency acoustic attenuation media has been significantly advanced by the integration of graphene derivatives and carbon nanotubes, taking advantage of their superior chemical and mechanical attributes.

## Graphical abstract

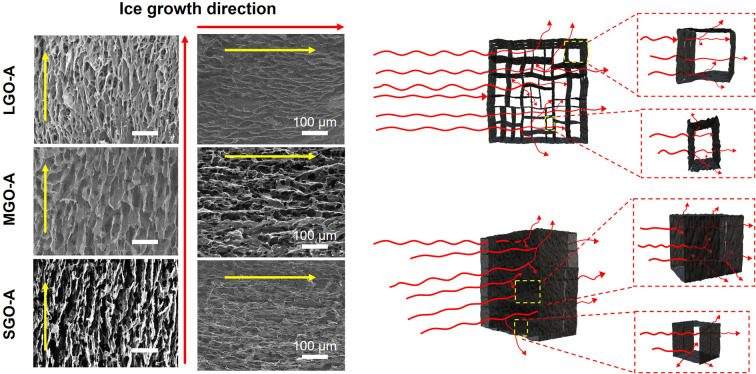

## 1. Introduction

In the disciplines of mechanical and materials engineering, there is a growing emphasis on the morphological regulation of cellular architecture through the precise engineering of hierarchical pore systems.^1,2^ These advanced frameworks are highly valued for their superior strength-to-weight ratios, elastic compressibility, and specialized wave attenuation capabilities. Nevertheless, achieving a successful synthesis of microporous hierarchical networks that incorporate multiple components remains a formidable task.^3,4^ This complexity arises from significant technical hurdles related to geometric constraints and the inherent physical properties of the constituent materials. Especially, while conventional optimization has focused on variables such as pore interconnectivity and cell diameter, the engineering of economical, high-performance acoustic cellular networks *via* structural manipulation remains a significant challenging issue. Also, achieving superior sound attenuation without increasing production costs requires a more sophisticated approach to hierarchical design.

Effective noise mitigation has become a critical imperative in modern industrial and urban landscapes, catalyzing the demand for next-generation functional acoustic absorption materials. Conventional porous media, such as polymer foams and mineral wools, are frequently constrained by excessive bulkiness and suboptimal attenuation in the low-frequency regime.^5–7^ Despite the potential for frequency tuning and enhanced absorption through phase manipulation in acoustic metamaterials, precise nanoscale engineering is required, which currently remains highly demanding and difficult to realize. Recently, graphene-based aerogels (GAs) have emerged as preeminent candidates for advanced shielding due to their ultra-low density, expansive specific surface area, and exceptional mechanical robustness.^8–10^ A key advantage of these carbon allotropes is their high thermal conductivity, which facilitates the rapid conversion of coherent pressure fluctuations into heat *via* viscothermal energy dissipation and isothermal heat exchange within the hierarchical porous network.^11–13^

The acoustic efficacy of an aerogel is intrinsically governed by its internal pore architecture, which determines the tortuosity and airflow resistivity encountered by propagating sound waves.^14,15^ While stochastic pore morphologies often exhibit limited absorption coefficients, directional freeze-casting (ice-templating) allows for the precise engineering of anisotropic pore alignments. Specifically, bidirectional freeze-casting enables the synthesis of highly ordered lamellar networks supported by a macroscopic skeleton.^16,17^ These structures can be strategically oriented in parallel or perpendicular configurations relative to the incident sound vector to optimize wave propagation paths and mitigate shearing resonances. While the exploration of synthetic pathways for anisotropic graphene aerogels remains in its preliminary stages, the unique attributes of micro-architectured cellular graphene oxide (GO) with highly ordered pore alignments demonstrate significant potential for visco-thermal energy dissipation. Although the development of graphene-based structures for sound mitigation has emerged as a prominent field of study, there is, to the best of our knowledge, no documented literature specifically addressing the anisotropic characteristics of pore-aligned reduced graphene oxide (rGO) structures in direct correlation with their frequency-dependent sound absorption mechanisms. While many studies have focused on composites, the systematic evaluation of how flake size and loading dictate the barrier effect and structural anisotropy within pristine graphene aerogels remains an open challenge in acoustic engineering.

In this study, we present a systematic framework to tailor the acoustic dissipation of reduced graphene oxide (rGO) aerogels by synergistically controlling flake dimensions and bidirectional orientation. In self-assembled architecture, the sheet dimensions significantly modulate the formation of lamellar walls and internal barriers (septa). While large GO sheets (LGO) facilitate the formation of continuous, streamlined channels with high aspect ratios, small GO sheets (SGO) promote a higher density of internal structural partitions. These partitions arise from the limited bridging capability of SGO during ice crystal growth, creating a compartmentalized micro-geometry that functions as a series of multiple scattering centers for incident waves. Furthermore, this study provides profound insights into the acoustic absorption characteristics arising from anisotropy-induced multiple scattering in both parallel and vertical graphene aerogels. The complex microcellular morphology, characterized by unidirectionally semi-open cells and aligned graphene oxide walls, provides an expansive effective surface area that generates substantial flow resistivity, thereby reducing the phase velocity of sound and maximizing the efficient dissipation of acoustic energy into thermal energy. By elucidating the structure–property relationships between anisotropic alignment and frequency-specific damping, this research provides new insights into the design of high-performance, multifunctional carbon-based noise insulators capable of outperforming commercial standards.

## 2. Experimental

### 2.1. Synthesis of graphene oxide

Graphene oxide was synthesized from graphite powder *via* a modified Hummer's method. Briefly, graphite powder was dispersed in concentrated H_2_SO_4_ under constant stirring within an ice bath to maintain the reaction temperature below 5 °C. KMnO_4_ was subsequently added in a slow, stepwise manner to facilitate the *in situ* generation of the powerful oxidant manganese heptoxide (Mn_2_O_7_) while preventing thermal runaway. The resulting mixture was then transferred to a 35 °C in oil bath and stirred to allow for the oxidative intercalation of the graphite layers. The oxidation process was further advanced by the gradual addition of deionized water, which induced a strong exotherm, raising the temperature to approximately 95 °C and shifting the suspension color to brownish yellow. To terminate the reaction, H_2_O_2_ (30%) was introduced to reduce residual permanganate and manganese dioxide into soluble MnSO_4_, resulting in a characteristic bright yellow slurry. The crude product was purified through multiple washing cycles involving HCl (10%) and deionized water *via* centrifugation until a neutral pH was achieved. The final graphene oxide was obtained by isolating the supernatant from any unreacted graphite precursors.

### 2.2. Fabrication of reduced graphene oxide aerogel (rGA)

The graphene oxide aerogel (GO-A) and its reduced counterpart (rGO-A) were fabricated using a directional freezing and chemical vapor reduction process. Initially, a cylindrical mold with a diameter of 29 mm was positioned on an aluminum plate. Graphene oxide (GO) dispersions of varying lateral sizes were poured into the mold and pre-cooled in a freezer for 10 min. To induce controlled ice crystal growth, the samples were transferred to a liquid nitrogen container for 30 min, ensuring no direct contact with the liquid nitrogen to maintain a stable freezing gradient. The frozen samples were subsequently subjected to lyophilization (freeze-drying) for 48 h to yield the porous rGO-A structure, after which their initial sound absorption coefficients were characterized.

### 2.3. Characterizations

XRD (MiniFlex 600, Rigaku) was conducted using a Cu radiation source at a scan rate of 5° min^−1^. Raman spectra were measured using a Raman spectrometer (FEX, NOST, Korea) with 532 nm laser excitation. XPS was performed using an X-ray photoelectron spectrometer (K-Alpha, Thermo Scientific) to analyze the chemical compositions of the samples. Morphologies and structures of the aerogel samples were examined by SEM (MiRA3-LMH, Tescan). TEM was conducted by JEOL JEM-F200 and FEI Tecnai G2 F30 electron microscopes operating at accelerating voltages of 200 and 100 kV, respectively. The normal incident sound absorption coefficient of all samples was characterized using a Brüel & Kjær impedance tube, type 4206. A cylindrical sample of 29 mm in diameter was used to measure the frequency band from 50 to 6.4 kHz *via* the two-microphone method. Airflow resistivity measurements based on ASTM C4522-03 were conducted in a laminar flow state (0.5 to 50 mm s^−1^).

### 2.4. Electrochemical measurements

Electrochemical performances of the electrodes were evaluated by the electrochemical workstation (BioLogic SP-150 Potentiostat) using a three-electrode system with a graphite rod as the counter electrode, Ag/AgCl as the reference electrode, and as-prepared rGO-A as the working electrode in H_2_SO_4_ electrolyte (0.5 M). Before the electrochemical tests, high-purity N_2_ was introduced into the electrolyte for 30 min. Polarization curves for HER were obtained *via* LSV at a scan rate of 5 mV s^−1^ with IR compensation.

## 3. Results and discussion

### 3.1. Size tailoring of graphene oxide: synthesis methods and physicochemical properties

**Fig. 1:**
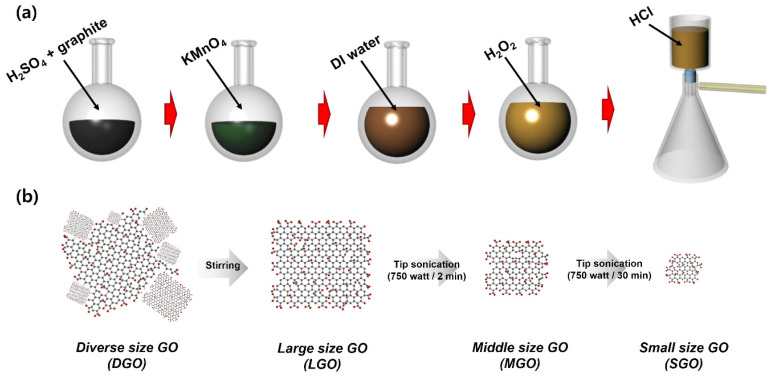
Schematic representation of the synthesis and size-tailoring of GO. (a) Synthesis of raw GO *via* the modified Hummers' method involving chemical oxidation and purification. (b) Sequential size-reduction process: extraction of LGO *via* sedimentation during stirring, followed by the fabrication of MGO (750 W, 2 min) and SGO (750 W, 30 min) through time-controlled tip sonication.

The systematic fabrication and lateral size tuning of graphene oxide (GO) are schematically delineated in Fig. 1. Initially, GO was synthesized from natural graphite flakes *via* a modified Hummers' method.^18^ As illustrated in Fig. 1a, the graphite underwent a rigorous chemical oxidation process in a concentrated H_2_SO_4_ and KMnO_4_ medium, followed by the addition of DI water and H_2_O_2_ to terminate the reaction. The resulting Diverse size GO (DGO) exhibited a highly polydisperse and stochastic size distribution, a common characteristic of bulk chemical exfoliation. To achieve precise morphological control, a multi-stage separation and mechanical fragmentation strategy was implemented (Fig. 1b). The raw DGO suspension was first subjected to a controlled stirring phase. By exploiting differential sedimentation rates, the relatively larger and more massive GO sheets were selectively isolated from the precipitated fraction, yielding large size GO (LGO). Subsequently, mechanical refinement was achieved through high-power tip sonication (750 W). Middle size GO (MGO) was obtained by subjecting LGO to 2 min of sonication, where high shear forces induced controlled fragmentation into intermediate scales. To reach the terminal minimum dimensions, the MGO was subjected to an extended 30 minute sonication period, resulting in the production of small size GO (SGO).

**Fig. 2:**
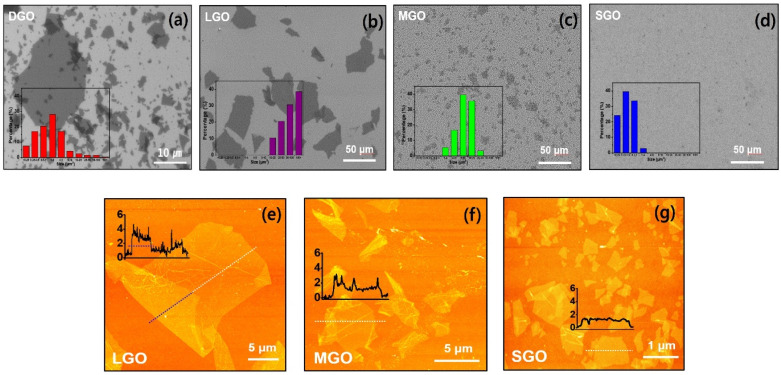
Morphological characterization and size distribution of GO samples. SEM images and corresponding histograms of (a) DGO showing uncontrolled sizes, (b) LGO (mostly >50 µm), (c) MGO (peak ∼ 10 µm), and (d) SGO (<1 µm). AFM topographical images and height profiles of (e) LGO, (f) MGO, and (g) SGO, confirming successful lateral size reduction while maintaining a sheet thickness below 2 nm.

The lateral dimensions and overall uniformity of the GO cohorts were quantitatively evaluated using SEM and corresponding statistical histograms in Fig. 2a–d. In Fig. 2a, the initial DGO reveals an uncontrolled and broad size distribution characteristic of the primary oxidation product. In contrast, the LGO sample comprises predominantly large-scale flakes exceeding 10 µm, with a significant majority surpassing the 50 µm threshold in Fig. 2b. In Fig. 2c and d, the MGO sample displays an intermediate distribution (1–10 µm) with a statistical peak centered at approximately 6 µm, while SGO reveals highly refined flakes reduced to sub-micrometer scales, consistently measuring below 1 µm. In Fig. 2e–g, further validation of the topographical properties was conducted *via* Atomic Force Microscopy (AFM). The LGO exhibited lateral dimensions of approximately 15 µm, with a height profile indicating a thickness of ∼4 nm at overlapping regions (denoted by the black dotted line) and a single-layer thickness below 2 nm in the primary regions.^19,20^ Also, both MGO and SGO maintained this consistent thickness of less than 2 nm despite the substantial reduction in lateral area. These results demonstrate that thin-layered GO was uniformly separated according to size.

**Fig. 3:**
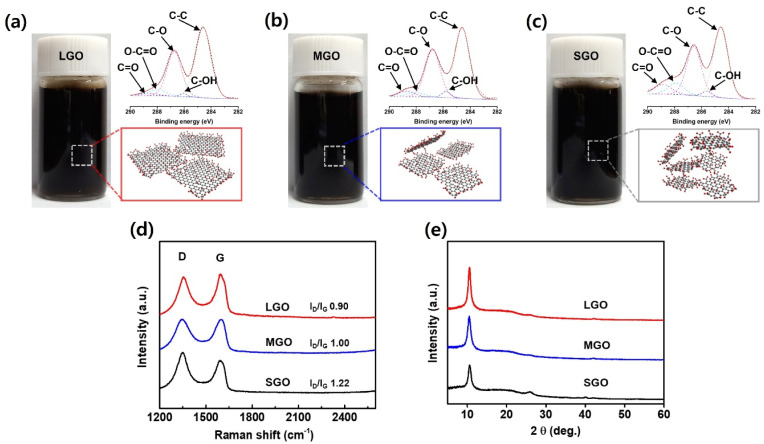
Characterization and structural analysis of Graphene Oxide (GO) samples with varying lateral sizes: Large GO (LGO), Medium GO (MGO), and Small GO (SGO). (a) Aqueous dispersion digital image and C 1s XPS analysis of LGO. (b) Aqueous dispersion digital image and C 1s XPS analysis of MGO. (c) Aqueous dispersion digital image and C 1s XPS analysis of LGO. (d) Raman spectra of LGO, MGO and SGO. (e) X-ray diffraction of LGO, MGO and SGO.

The chemical functionality and crystalline integrity of the size-controlled GO were investigated using XPS, Raman, and XRD analyses in Fig. 3. The digital images of LGO, MGO and SGO show a highly stable and uniform dispersion in water, exhibiting a characteristic dark brown color indicative of well-exfoliated graphene oxide. The stability of the dispersions was confirmed through time-dependent UV-Vis analysis, where the concentrations of LGO (0.6 mg mL^−1^), MGO (0.7 mg mL^−1^), and SGO (0.12 mg mL^−1^) remained consistent over time, indicating excellent colloidal stability in Fig. S1. The C 1s XPS spectra for LGO, MGO, and SGO confirm successful functionalization with oxygen-bearing moieties, specifically epoxy (C–O; 284.5 eV), hydroxyl (C–OH; 285.5 eV), carbonyl (C=O; 288.2 eV) and carboxyl (O–C=O; 290.1 eV) groups in Fig. 3a–c.^21,22^ Notably, due to the increased specific surface area inherent in smaller flakes, the oxygen content exhibits a progressive increase as lateral size decreases in Table S1. Fig. 3d presents structural evolution was further examined *via* Raman spectroscopy. All samples displayed characteristic D and G bands; however, the ID/IG ratio rose from 0.90 for LGO to 1.00 for MGO, reaching a maximum of 1.22 for SGO. This enhancement in the ID/IG ratio correlates with the increased density of edge-induced defects and higher surface-to-volume ratios as the sheets are fragmented. Finally, the XRD patterns for all specimens revealed a prominent (001) reflection At a 2*θ* value of approximately 10.6°, where the broad nature of these peaks is indicative of the random stacking morphology of the GO sheets in Fig. 3e.^23,24^ The multifaceted analyses presented in this study confirm a highly reliable process for precisely controlling the size of GO while preserving its chemical properties and ultra-thin layered structure.

### 3.2. Microstructural characterization of aligned GO aerogels

**Fig. 4:**
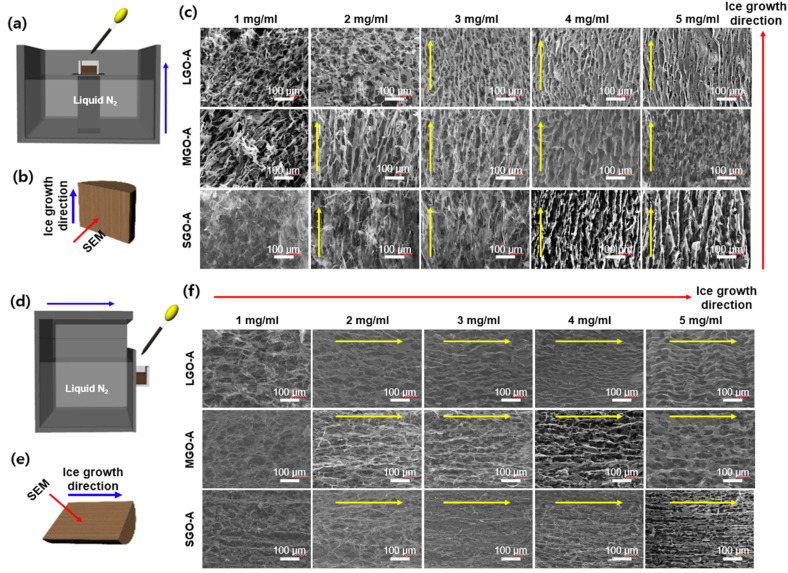
Microstructural characterization of vertically aligned GO aerogels prepared from LGO, MGO, and SGO at various concentrations *via* bidirectional freezing. (a) Schematic illustration of the GO aerogel fabrication process using a bidirectional freezing technique. (b) Schematic of GO growth direction and SEM observation sites. (c) SEM images of GO aerogels: SEM images showing the cross-sectional morphologies of LGO-A, MGO-A, and SGO-A *via* concentration. Microstructural characterization of horizontally aligned GO aerogels prepared from LGO, MGO, and SGO at various concentrations *via* bidirectional freezing. (d) Schematic illustration of the GO aerogel fabrication process using a bidirectional freezing technique. (e) Schematic of GO growth direction and SEM observation sites. (f) SEM images of GO aerogels: SEM images showing the cross-sectional morphologies of LGO-A, MGO-A, and SGO-A *via* concentration.

The architectural design of vertical aligned, anisotropic graphene oxide (GO) frameworks was realized through a sophisticated bidirectional freeze-casting methodology. As schematically delineated in Fig. 4a, GO aqueous suspensions were subjected to a precisely modulated thermal gradient within a cryogenic liquid nitrogen environment. This setup induces controlled nucleation and the unidirectional propagation of ice crystals, which serve as a sacrificial template.^25,26^ During this ice-segregation-induced self-assembly (ISISA) process, GO flakes are excluded from the advancing planar solidification front and subsequently sequestered within the interstitial spaces between crystals, effectively forming a cohesive and vertically oriented monolithic structure. Fig. 4b provides a conceptual visualization of the resulting aerogel, where the structural orientation demonstrates high directional fidelity parallel to the primary ice growth vector.

To maintain precise stoichiometric ratios and consistent experimental outcomes, the concentration of each GO precursor suspension was rigorously verified *via* UV-Vis spectrophotometry, adhering to the Beer–Lambert behavior, demonstrating the linear correlation between absorbance and concentration.^27^*A* = *ε* × *c* × *l**A*: absorbance, *ε*: molar absorptivity, *c*: concentration, *l*: length.

Quantitative spectroscopic analysis confirmed a linear correlation between optical density and mass concentration. Intriguingly, smaller GO flakes (SGO) exhibited a high absorption coefficient compared to their larger counterparts (LGO) at equivalent mass loadings—a phenomenon attributed to the size-dependent scattering and absorption cross-sections inherent in low-dimensional carbon allotropes in Fig. S2. The evolution of the internal pore architecture as a function of lateral flake dimensions and mass concentration (ranging from 1 to 5 mg mL^−1^) was comprehensively evaluated through cross-sectional SEM in Fig. 4c. At lower loading regimes (1–2 mg mL^−1^), the insufficient packing density of the GO flakes fails to overcome Brownian motion and hydrodynamic forces during ice growth, resulting in the formation of predominantly isotropic and disordered porous networks. However, a critical transition toward a highly ordered bidirectional morphology is observed at concentrations exceeding 3 mg mL^−1^, characterized by the emergence of well-defined, continuous lamellar walls. At the optimal concentration of 4 mg mL^−1^, superior structural anisotropy was observed across all samples, including LGO-A, MGO-A, and SGO-A. A comparative analysis reveals that the lateral size of the constituent flakes plays a decisive role in governing the internal micro-geometry. In case of LGO-A, the resulting framework exhibits a highly aligned channel structure by the high aspect ratio and expansive surface area of LGO flakes. These channels are characterized by long-range continuity and a remarkable absence of transverse obstructions, facilitating unimpeded transport pathways. In SGO-A, conversely, the reduced lateral dimensions of SGO flakes promote the formation of frequent internal structural bridges or barriers. This leads to a compartmentalized bidirectional architecture where the primary vertical channels are intermittently punctuated by transverse GO partitions, effectively increasing the structural complexity and tortuosity of the network. At the supra-optimal concentration of 5 mg mL^−1^, the excessive solid loading leads to a highly congested and clogged framework. This overcrowded state manifests as a dense bidirectional structure with a high frequency of structural blockages, which may significantly attenuate the permeability and functional transport properties of the aerogel. By elucidating the morphological evolution of bidirectional frameworks relative to precursor size and concentration, this study demonstrates that the synergy between microscopic dimensions and macroscopic rheology dictates the hierarchical tailoring of anisotropic carbon-based monoliths.

The internal architecture and transverse symmetry of the fabricated graphene oxide (GO) aerogels were systematically investigated *via* analysis of horizontal cross-sections, oriented perpendicular to the primary ice growth vector. The methodology of these horizontally oriented bidirectional architectures is schematically elucidated in Fig. 4d. Also, Fig. 4e provides a conceptual visualization of the resulting monolithic structures from a transverse perspective. The influence of lateral flake size (LGO, MGO, and SGO) and mass concentration (ranging from 1 to 5 mg mL^−1^) on the internal pore geometry was rigorously evaluated through SEM analysis as shown in Fig. 4f. Consistent with the longitudinal observations in Fig. 4c, specimens prepared at low loading regimes (1 and 2 mg mL^−1^) failed to establish long-range structural order, whereas a distinct transition toward a highly ordered bidirectional morphology was observed with increasing concentration. In agreement with the preceding findings, the morphology evolves from elongated, uninterrupted lamellar channels induced by large flakes to a compartmentalized and tortuous network with smaller flakes, while at supra-optimal concentrations, excessive wall thickness and structural blockages compromise the framework, resulting in a diminished effective porosity.

The chemical reduction of size-controlled GO aerogels was strategically executed to restore the extended sp^2^ conjugated carbon network, a critical requirement for facilitating efficient charge carrier transport and enhancing both electrical and thermal conductivities.^28–30^ Fig. S3a presents digital macro-graphs of the reduced LGO, MGO, and SGO (rLGO, rMGO, and rSGO) aerogel monoliths across a concentration gradient from 1 to 5 mg mL^−1^, confirming that the hierarchical structural integrity and macroscopic uniformity remain intact post-reduction. The morphological integrity of the GO aerogels remained largely unchanged following the reduction treatment in Fig. S4. The electrical transport properties of these 3D frameworks were systematically characterized, as depicted in Fig. S3b. A robust inverse correlation exists between mass concentration and electrical resistance; specifically, augmenting the solids loading significantly enhances electrical conductivity by increasing the network density. rLGO aerogels consistently exhibit the highest conductivity, which is attributed to the reduction of inter-sheet junction resistance and a lower density of contact points within the expansive flake network. Intriguingly, at a concentration of 5 mg mL^−1^, as the specimens reach the percolation threshold, the high degree of interconnection within the conductive pathways nullifies the influence of initial flake size on the overall electrical behavior. Fig. S5 reveals that the rLGO-A, rMGO-A, and rSGO-A variants maintain a turbostratic, random-stacking morphology, as evidenced by the characteristic broad diffraction peaks observed in XRD spectra. To evaluate the effectiveness of the increased conductivity in functional applications, the aerogels were evaluated for their electrocatalytic activity toward the Hydrogen Evolution Reaction (HER) using a standard three-electrode configuration in 0.5 M H_2_SO_4_ in Fig. S3c. The polarization curves obtained *via* Linear Sweep Voltammetry (LSV) in Fig. S3d demonstrate that HER performance is intrinsically coupled to the conductivity and structural anisotropy of the aerogels. Higher precursor concentrations lead to a substantial reduction in the onset overpotential, reflecting improved charge-transfer kinetics. The rGO-A electrode exhibits a superior overpotential compared to the graphite sheet; notably, the overpotential further improves as the electrical conductivity increases. The enhanced activity confirms the optimization of charge–transfer pathways facilitated by the high electrical and thermal conductivity of rGO-A; such structural refinements exert a profound influence on the resulting sound absorption characteristics.

### 3.3. Acoustic absorption of aligned GO aerogels

**Fig. 5:**
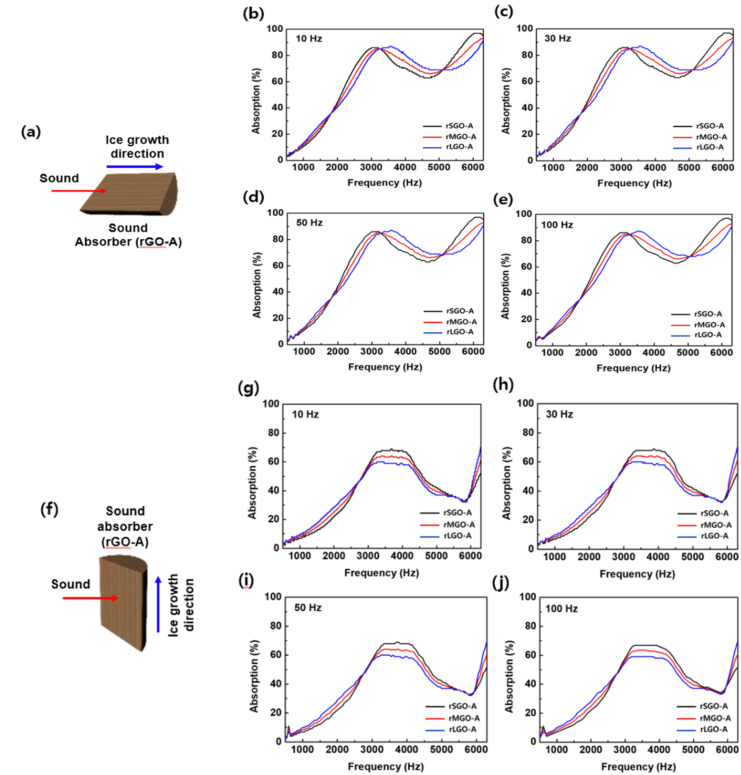
Comparison of the sound absorption coefficient between rLGO-A, rMGO-A and rSGO-A. (a) The image of the orientation of the sound absorber and the horizontal incidence of sound. (b) The sound absorption coefficient of rGO-A at an incident sound frequency of 10 Hz. (c) The sound absorption coefficient of rGO-A at an incident sound frequency of 30 Hz. (d) The sound absorption coefficient of rGO-A at an incident sound frequency of 50 Hz. (e) The sound absorption coefficient of rGO-A at an incident sound frequency of 100 Hz. Comparison of the sound absorption coefficient between rLGO-A, rMGO-A and rSGO-A. (f) The image of the orientation of the sound absorber and the vertical incidence of sound. (g) The sound absorption coefficient of rGO-A at an incident sound frequency of 10 Hz. (h) The sound absorption coefficient of rGO-A at an incident sound frequency of 30 Hz (i) The sound absorption coefficient of rGO-A at an incident sound frequency of 50 Hz. (j) The sound absorption coefficient of rGO-A at an incident sound frequency of 100 Hz.

The acoustic attenuation characteristics of the hierarchically engineered rGO aerogels were systematically investigated by evaluating the sound absorption coefficient (*α*) across a comprehensive frequency spectrum. As depicted in Fig. 5a, all monolithic specimens were synthesized at an optimized precursor concentration of 4 mg mL^−1^ to ensure structural robustness and long-range anisotropic order. To evaluate their acoustic response, incident sound waves were applied to both horizontally and vertically grown rGO aerogels at discrete frequencies of 10, 30, 50, and 100 Hz. This configuration facilitates a direct correlation between the directionally engineered bidirectional micro-channels and the resulting viscothermal energy dissipation mechanisms.^31,32^Fig. 5b–e shows the absorption profiles as a function of incident frequencies at 10, 30, 50, and 100 Hz. The normal incidence sound absorption coefficients of all samples were obtained using the Brüel & Kjær impedance tube. The absorption data reveals that all size-tailored aerogels exhibit exceptional acoustic dissipation efficiency, with absorption peaks exceeding 80% within the 3000–3500 Hz bandwidth irrespective of the incident sound frequency. Notably, the internal micro-geometry significantly modulates the frequency-specific response through distinct energy dissipation pathways. rSGO-A (Small-size GO Aerogel) demonstrates marked enhancement in the lower frequency regime (1000–3000 Hz). This optimized low-frequency performance is intrinsically linked to the high density of internal structural barriers, which increase the tortuosity of the air-pore interface and amplify energy dissipation *via* multi-path scattering and intensified interfacial friction. Conversely, the rLGO-based architecture—characterized by its streamlined, barrier-free lamellar channels—exhibits superior acoustic attenuation in the high-frequency domain (>4000 Hz), where the unobstructed pore geometry facilitates efficient dissipation of short-wavelength waves.

The orientation-dependent acoustic attenuation properties were further characterized by aligning the incident sound vector perpendicular to the longitudinal ice growth direction in Fig. 5f. This setup establishes a distinct contrast to the longitudinal alignment, elucidating the profound structural anisotropy inherent in these frameworks. A clear performance hierarchy was established among the size-tailored samples, with rSGO-A exhibiting the highest absorption efficiency, followed by rMGO-A and rLGO-A, regardless of the frequency of the incident sound in Fig. 5g–j. Specifically, rSGO-A exhibits the most prominent absorption peaks, attaining approximately 70% efficiency within the 3500–4000 Hz bandwidth. In this transverse orientation, the elevated density of internal structural barriers functions as multiple scattering centers, significantly amplifying energy dissipation *via* intensified interfacial friction and enhanced viscothermal losses. In contrast, rLGO-A demonstrates relatively diminished absorption efficiency; the paucity of internal barriers within its streamlined lamellar channels leads to attenuated wave–structure interactions when acoustic energy is incident across the pore walls. While rSGO-A remains the superior absorber, the overall magnitude in this transverse mode is moderately reduced compared to the longitudinal mode, suggesting that the influence of structural barriers is partially mitigated when sound waves are incident perpendicular to the channels. These observations underscore the highly anisotropic nature of acoustic dissipation in rGO aerogels, allowing the acoustic response to be strategically engineered by modulating precursor flake dimensions and spatial orientation.^33^ Furthermore, to evaluate the sound-absorbing efficiency of the acoustic specimens, the Noise Reduction Coefficient (NRC) was determined, with results presented in Fig. 5a. Representing a critical benchmark for damping materials, the NRC was derived by averaging the sound absorption coefficients across four specific 1/3 octave bands: 250, 500, 1,000, and 2000 Hz. The sound absorption properties of rSGO-A exhibited a slight enhancement compared to those of rMGO-A and rLGO-A. More importantly, the samples oriented in the parallel direction demonstrated superior acoustic performance compared to their perpendicular counterparts in Fig. S6.

### 3.4. Acoustic absorption mechanism of aligned GO aerogels

**Fig. 6:**
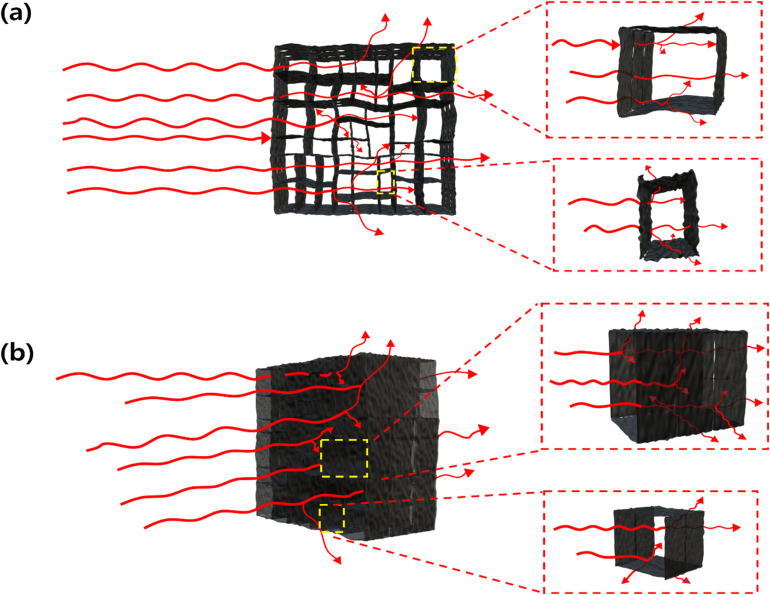
Schematic illustration of the sound absorption mechanism within a directionally antagonistic graphene absorber: (a) wave reflection in perpendicular rGO-A and (b) wave absorption in parallel rGO-A.

Up to this point, this study investigates the effects of diverse aerogel architectures, tailored by the concentration, size, and orientation of graphene oxide (GO), on their resulting sound absorption coefficients. Our focus now shifts to elucidating the acoustic absorption mechanisms within the directionally antagonistic rGO-A. Fundamentally, sound absorption involves the dissipation of acoustic energy, where the kinetic energy of medium particles is eventually converted into thermal energy.^34^ In rGO-A, this attenuation occurs through thermal and viscoelastic frame damping, increased tortuosity along the graphene oxide surfaces, and heat-extracting microvibrations within the narrow graphene layers.^35^ As illustrated in Fig. 6a, sound waves traversing the perpendicular rGO-A undergo a series of reflections and transmissions across the graphene layers. Furthermore, as these waves penetrate the interstices between the graphene oxide walls, pressure fluctuations trigger microvibrations in the aligned layers. Conversely, Fig. 6b demonstrates the absorption principle within the internal voids of parallel rGO-A. In this configuration, extensive multiple scattering significantly diminishes the acoustic intensity within the highly porous foam, markedly enhancing absorption. This pressure attenuation stems from viscous friction and thermal conduction within the surrounding graphene oxide microstructure.^36^ From a viscoelastic damping perspective, molecular friction within the solid domain transforms vibrational energy into heat loss. This process is highly efficient because the structure, consisting solely of graphene, facilitates the rapid dissipation of generated heat. Structurally, the aligned graphene frame creates elongated pathways that increase tortuosity and scattering. Ultimately, as sound waves reach the narrow gaps between layers, the resulting air oscillation leads to significant energy loss through friction and layer microvibration.

## 4. Conclusion

In summary, this research successfully demonstrates a systematic strategy for engineering high-performance anisotropic reduced graphene oxide aerogels (rGO-A) with frequency-tunable acoustic absorption. By precisely modulating the lateral flake dimensions of the graphene oxide (GO) precursors and employing a bidirectional freeze-casting methodology, we fabricated highly ordered, vertically and horizontally aligned lamellar networks. Morphological investigations established that the microscopic dimensions of the GO sheets are paramount in determining the macroscopic hierarchical architecture. While large flakes (LGO) facilitate the formation of continuous, streamlined micro-channels with minimal structural resistance, smaller flakes (SGO) induce a high density of internal structural barriers, or septa; this fragmented microstructure stems from the insufficient bridging capability of SGO during ice templating, which leads to the development of internal partitions. A clear hierarchy in acoustic performance was identified, with rSGO-A exhibiting superior absorption characteristics compared to rMGO-A and rLGO-A at low-to-mid frequencies, thereby validating the impact of microscopic architecture on macroscopic properties. This enhanced efficacy is driven by intensified visco-thermal energy dissipation and multiple scattering effects facilitated by the increased tortuosity and internal air-flow resistance provided by the dense septa. Furthermore, the study validated the profound structural anisotropy of these frameworks, which induces extensive multiple scattering within the internal voids of parallel rGO-A, leading to superior absorption characteristics compared to vertical rGO-A. This configuration substantially attenuates acoustic intensity as waves traverse the highly porous foam, leading to a significant enhancement in overall absorption performance. These findings provide a new design paradigm for the synthesis of lightweight, multifunctional carbon-based noise insulators, offering a scalable solution that outperforms commercial standards in frequency-specific damping applications.

## Author contributions

The manuscript was written with contributions from all authors. S. H. S. and D. Lee conceptualized the experiments and wrote the manuscript. Writing—original draft preparation, S. H. S. writing—review and editing. All authors contributed to the data analysis and discussion of the results. All authors have given approval of the final version of the manuscript. All authors have read and agreed to the published version of the manuscript.

## Conflicts of interest

The authors declare no conflicts of interest.

## Supplementary Material

RA-016-D6RA01470D-s001

## Data Availability

The data supporting this article have been included as part of the supplementary information (SI). Supplementary information (SI) is available. See DOI: https://doi.org/10.1039/d6ra01470d.
