# Supplementary material for: Frequency-tunable acoustic absorption in anisotropic graphene aerogels via morphological engineering of internal barriers
Source: RSC Adv. 2026 May 12;16(27):24893–902. doi: 10.1039/d6ra01470d (PMC13161953; doi:10.1039/d6ra01470d)
Supplement: RA-016-D6RA01470D-s001 [file RA-016-D6RA01470D-s001.pdf]

Supplementary Information (SI) for RSC Advances.
This journal is © The Royal Society of Chemistry 2026

Supporting Information

Frequency-Tunable Acoustic Absorption in Anisotropic Graphene Aerogels via Morphological Engineering of Internal Barriers

Jaeeun Jin[1,†], Jae Gyu Ahn[1,†], An So yeon[1], Ryunkyong Lee[1], Taeyoung Park[1], Dong Ju Lee[2,*], Sung Ho Song[1,*]

*Dong Ju Lee
E-mail: dongjulee@chungbuk.ac.kr

*Sung Ho Song
E-mail: shsong805@kongju.ac.kr

[1] Division of Advanced Materials Engineering, Center for Advanced Materials and Parts of Powders, Kongju National University, Cheonan-si 31080, Republic of Korea

[2] Department of Urban, Energy, and Environmental Engineering, Chungbuk National University, Cheongju 28644, Republic of Korea

Supporting Information Table S1

Table S1. C1s XPS Analysis of LGO, MGO and SGO.

	L-GO	*M-GO*	*S-GO*
**C-C/C=C**	***57.35%***	***53.04%***	***47.66%***
**C-OH**	***1.20%***	***2.75%***	***2.92%***
**C-O**	***34.15%***	***35.82%***	***36.72%***
**C=O**	***5.12%***	***5.45%***	***7.29%***
**O-C=O**	***2.18%***	***2.94%***	***5.41%***

Supporting Information Fig. S1

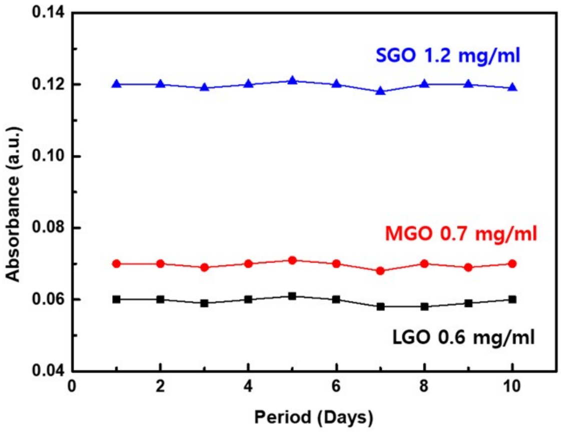

Fig. S1. Time-dependent UV-Vis analysis of LGO, MGO, and SGO dispersions at fixed concentrations of 0.6 mg/mL, 0.7 mg/mL, and 0.12 mg/mL, respectively.

Supporting Information Fig. S2

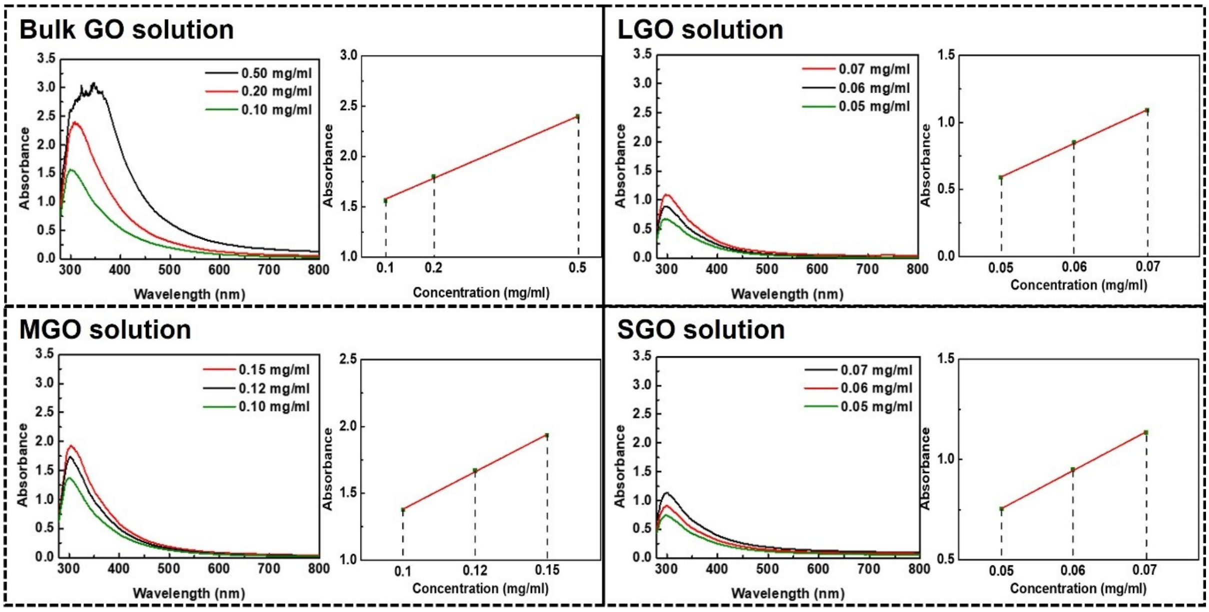

Fig. S2. UV-Vis Analysis of LGO, MGO and SGO as a function of concentration in water showing Beer Lambert behavior.

Supporting Information Fig. S3

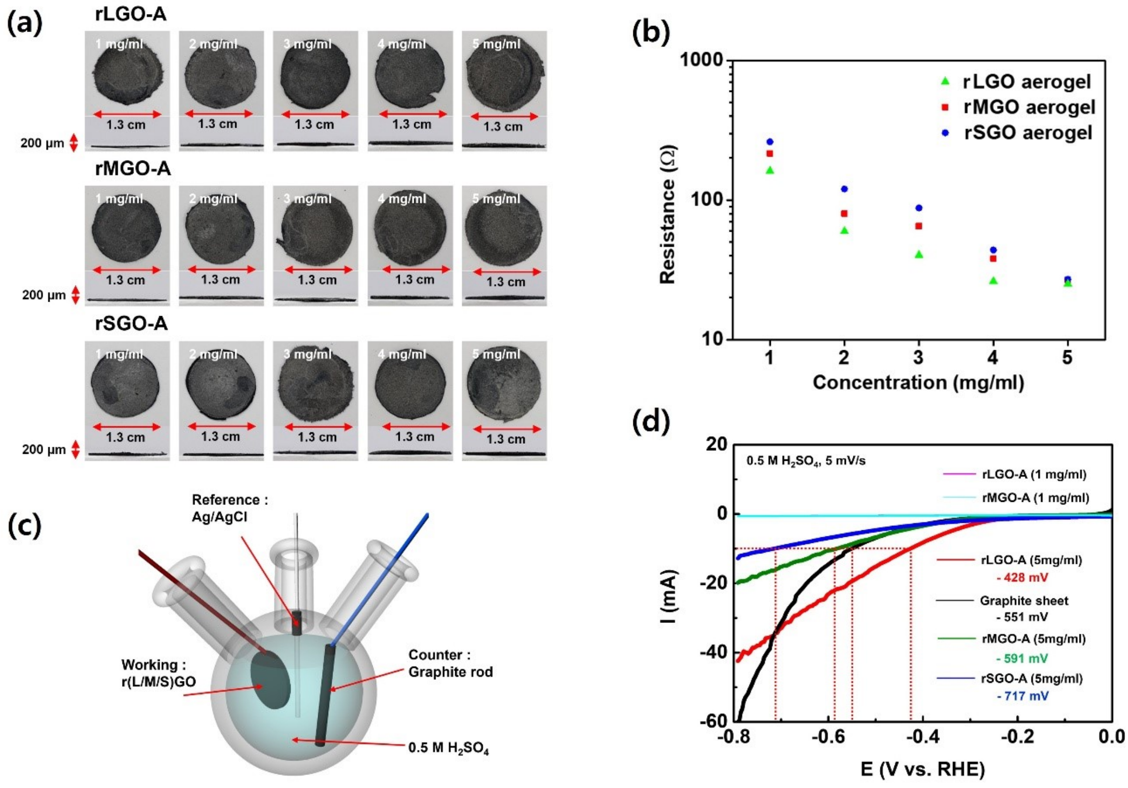

Fig. S3. Electrochemical Performance and Structural Properties of Reduced Graphene Oxide Aerogels (rGO-A). (a) The digital images of rLGO-A, rMGO-A, and rSGO-A via concentration (b) Characterization of electrical resistance of rLGO-A, rMGO-A, and rSGO-A via concetration. (c) Schematic illustration of the three-electrode system for HER (Hydrogen Evolution Reaction) measurements. (d) Hydrogen Evolution Reaction (HER) characteristics for rLGO-A, rMGO-A, and rSGO-A electrodes.

Supporting Information Fig. S4

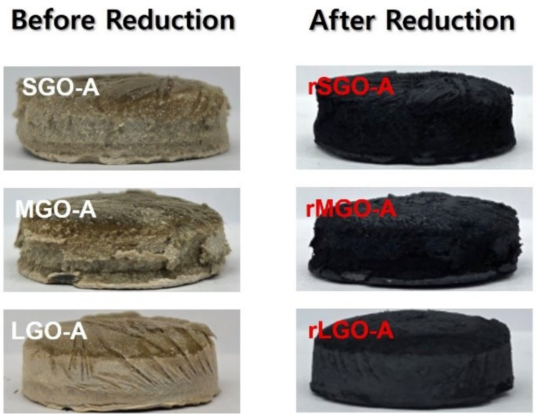

Fig. S4. Digital images of SGO-A, MGO-A, and LGO-A before and after reduction.

Supporting Information Fig. S5

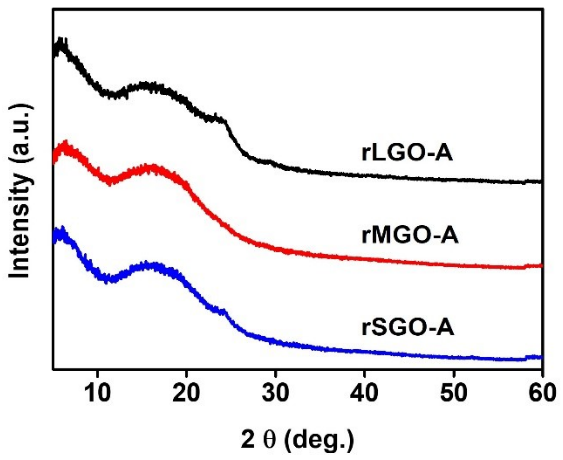

Fig. S5. X-ray diffraction of rLGO-A, rMGO-A and rSGO-A.

Supporting Information Fig. S6

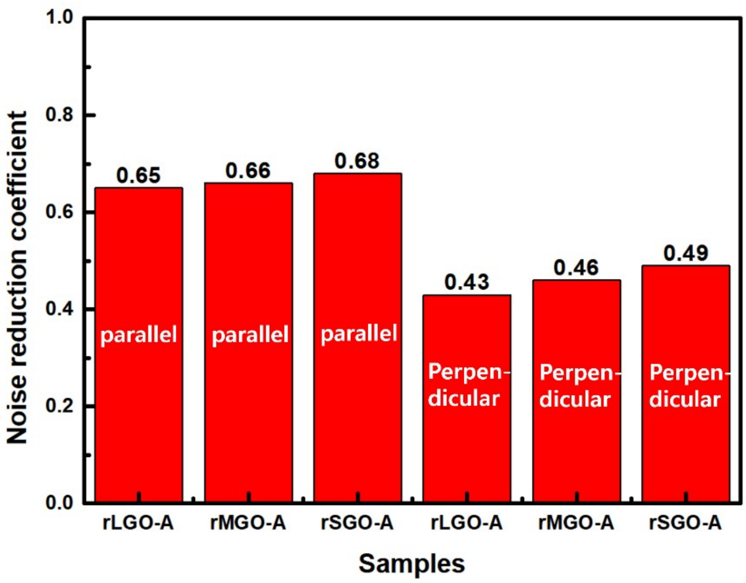

Fig. S6. Comparison of NRC(Noise reduction coefficient) values of the Parallel rGO-A and the Perpendicular rGO-A
